# Evaluation of Combined Disinfection Methods for Reducing *Escherichia coli* O157:H7 Population on Fresh-Cut Vegetables

**DOI:** 10.3390/ijerph120808678

**Published:** 2015-07-23

**Authors:** Eva Petri, Mariola Rodríguez, Silvia García

**Affiliations:** R&D&I Area, National Centre for Food Technology and Safety (CNTA), San Adrian 31570, Navarra, Spain; E-Mails: mrodriguez@cnta.es (M.R.); sgdelatorre@cnta.es (S.G.)

**Keywords:** fresh-cut vegetables, food sanitizers, decontamination, hurdle technology, microbiological quality, *Escherichia coli*, process water, peroxyacetic acid, chlorine dioxide

## Abstract

Most current disinfection strategies for fresh-cut industry are focused on the use of different chemical agents; however, very little has been reported on the effectiveness of the hurdle technology. The effect of combined decontamination methods based on the use of different sanitizers (peroxyacetic acid and chlorine dioxide) and the application of pressure (vacuum/positive pressure) on the inactivation of the foodborne pathogen *E. coli* O157:H7 on fresh-cut lettuce (*Lactuca sativa*) and carrots (*Daucus carota*) was studied. Fresh produce, inoculated with *E. coli* O157:H7, was immersed (4 °C, 2 min) in tap water (W), chlorine water (CW), chlorine dioxide (ClO_2_: 2 mg/L) and peroxyacetic acid (PAA: 100 mg/L) in combination with: (a) vacuum (V: 10 mbar) or (b) positive pressure application (P: 3 bar). The product quality and antimicrobial effects of the treatment on bacterial counts were determined both in process washing water and on fresh-cut produce. Evidence obtained in this study, suggests that the use of combined methods (P/V + sanitizers) results in a reduction on the microorganism population on produce similar to that found at atmospheric pressure. Moreover, the application of physical methods led to a significant detrimental effect on the visual quality of lettuce regardless of the solution used. Concerning the process water, PAA proved to be an effective alternative to chlorine for the avoidance of cross-contamination.

## 1. Introduction

Fresh-cut products are frequently involved in food disease outbreaks due to the fact that they are ready to eat, and are not subjected to further microbial killing steps before consumption. Therefore, the use of effective sanitizing agents during washing to ensure product safety is gaining more interest. 

However, it should be highlighted that some bacteria are attached/internalized in sites (like the stomata, crevices, *etc*.) inaccessible for these sanitizers, limiting their effectiveness [1,2,3,4]. The hydrophobic nature of vegetable surface also protects microorganisms from exposure to chemicals, which cannot penetrate into these natural openings on vegetable tissue. Therefore, it is clear that more work into this area is required, in order to improve the efficacy of pathogen reduction by means of understanding the mechanisms by which attached bacteria resist detachment [5]. Thus, an innovative strategy based on hurdle technology (positive or negative pressures + sanitizers in a simultaneous application) was explored for its potential to inactivate pathogens from the surface of fresh-cut produce by enhancing the contact between antimicrobial solution and bacteria. It was reported that lettuce leaves are covered by waxy surface cuticle layers and, thus, hydrophobic interactions should be the main forces affecting the bacterial attachment [6]. Use of positive and negative pressures could help in the disruption of such interactions, thereby improving the penetration of sanitizers when used in combination.

Different chemical washing agents have been studied to determine their efficacy in the inactivation of pathogenic bacteria on vegetables [7,8,9]. Although chlorine is still the most commonly used sanitizer due to its efficacy, cost-effectiveness ratio and simple use, future regulatory restrictions are likely and will require the development of functional alternatives. In some European countries including Germany, the Netherlands, Switzerland and Belgium, the use of chlorine in fresh-cut products is forbidden [10,11], due to the environmental and health risks posed. As a consequence, technically and economically effective chlorine-alternative disinfection technologies represent the main goal of the fresh-cut industry. 

Peroxyacetic acid or peracetic acid (PAA) has been proposed as sanitizing agent for its usefulness in the fresh-cut industry [12]. It is known as a strong oxidant agent, and it is commercially available as a mixture containing acetic acid, hydrogen peroxide and PAA, and its use forms an environmentally friendly acetic acid as only DBPs [13]. Moreover, the use of peroxyacetic has not been associated with the formation of disinfection byproducts [14]. Chlorine dioxide (ClO_2_) has also attracted interest as an alternative to chlorine, because its efficacy is less affected by pH and organic matter [15]. Unlike chlorine, chlorine dioxide does not participate in chlorination reactions that result in harmful byproducts, like trihalomethanes (THMs).

The scope of this study was to investigate the influence of the combination of physical methods (application of positive and negative pressures) simultaneously with alternative sanitizers(peroxyacetic acid, chlorine dioxide) on the decontamination performance, with the purpose of improving the exposure of potential pathogens, located in inaccessible sites on products, to sanitizing agents by removing gas or liquid barriers that block penetration of these sanitizers. These two vegetable species, iceberg lettuce and carrots, were included because they are representatives of different product types (leafy and root vegetables, respectively), different topologies of the vegetable tissue, besides of their economical relevance in the fresh-cut industry. Additionally, the efficacy of these sanitizing combinations to avoid cross-contamination in the processing water was also determined. Bearing in mind that the organic content of the wash water can influence the results, the conditions of wash water in commercial processing were simulated.

## 2. Materials and Methods

### 2.1. Bacterial Strain and Inoculation Procedure

*E. coli* O157:H7 obtained from the Spanish Type Culture Collection (CECT) was used in this study as a reference strain. This strain is non-pathogenic and therefore devoid of the ability to produce verotoxins. However, it possesses similar phenotypic characteristics with the toxigenic strain of *E. coli* O157:H7. Bacterial cultures were prepared in beads and kept in vials in a freezer at −80 ºC. The stock cultures were re-activated by inoculation onto Tryptic Soya Agar (TSA) + 0.6% yeast extract (YE) plates, which were incubated at 37 ºC for 24 hours.

Iceberg lettuce (*Lactuca sativa* var. capitata) and carrots (*Daucus carota*) were supplied by a Spanish fresh-cut vegetables processing company. After its reception, the produce was kept at 4 ºC until processing. The three to four external leaves of the lettuce were removed and manually cut into 3 × 3 cm pieces. All carrots were pre-cleaned, knife-peeled and topped. Afterwards, they were shredded using a multi-purpose cutting machine (model Fresh Express+, Moulinex). Fresh-cut iceberg lettuce and shredded carrots were divided in batches of 500 g per each washing treatment.

To inoculate the product, vegetables were immersed in the inoculum solution (water containing 9 log CFU/mL of *E. coli* O157:H7) and kept under constant agitation for 5 min. After dipping, excess liquid was removed for 30s in a manual salad spinner. The samples were placed into plastic containers and maintained at 37 ºC for 1 hour to favor adhesion of bacteria to vegetable tissue before treatment. A sample of the inoculated vegetable was analyzed to determine the initial *E. coli* O157:H7 concentration.

### 2.2. Preparation of Treatment Solutions

Processing water usually contains high organic load which, in most of the cases, reduces the efficacy of sanitizing agents and adversely affects quality and safety of fresh-cut products. In order to simulate commercial practices, process wash water used in this study was standardized. The chemical oxygen demand (COD) method was chosen to estimate the organic matter content of the process water as an indirect measure. Vegetables were placed into a blender (ECRON, model BH-3381) in order to obtain lettuce and carrot juices. Subsequently, the vegetable juice was diluted with tap water to obtain standardized process water. This process resulted in process water with a specific COD level (≈200 and 600 mg O_2_/L for lettuce and carrot, respectively).

The following sanitizing treatments were evaluated for efficacy in killing or reducing *E. coli* O157:H7 on lettuce and shredded carrots: a peroxyacetic acid based sanitizer (PAA) (Citrosol S.A., Valencia, Spain) at a concentration of 100 mg/L and chlorine dioxide (CLODOS PURO, STC Spain) at a concentration of 2 mg/L, based on the manufacturers’ recommended doses. A chlorinated water (CW) solution was produced containing 65 mg/L free chlorine by diluting sodium hypochlorite (Leon, commercial bleach, Spain with 35 g of active chlorine per liter) and standardized tap water. The pH of the washing solution was adjusted to 6.5 using citric acid to assure the presence of chlorine in the hypochlorous acid form in order to improve chlorine disinfection efficacy. Both tap water and chlorinated water were used as controls.

For the performance of the physical experiments, a vacuum system (Gastrovac, Selecta, Barcelona, Spain) and a positive pressure system (PC-20 Bachiller, Barcelona, Spain) were used. A container hermetically sealed was used for the experiments. In addition, a pressure gauge was used to measure pressure values: A load of 10 mbar was applied for the vacuum pressure and 3 bar for the positive pressure conditions for 2 min. After a negative or positive pressure period, the atmospheric pressure was restored in the tank.

For each washing treatment, one batch of vegetable pieces was immersed in process wash water containing the sanitizing solution for 2 minutes at 4 ºC in a 1:10 (w:v) product-water ratio.After washing, they were rinsed with tap water and centrifuged to remove excess of water prior to packaging.

### 2.3. Analytical Procedures

#### 2.3.1. Microbiological Analysis

Microbiological analyses were carried out before the disinfection step and immediately after processing. Microbial counts were performed by taking 10 g of fresh cut lettuce obtained from three different bags. They were placed aseptically in stomacher bags, diluted 1:10 in buffered peptone water (BPW) and homogenized by using a stomacher. Samples were plated in the appropriate culture media (TSA + YE) (Scharlau, Barcelona, Spain) and incubated at 37 ºC for 24 ± 4 h. After incubation, samples were enumerated and the microbiological counts were expressed as log_10_ colony forming units per gram (log CFU/g). The water samples were analyzed using the same enumeration methods and were expressed as log_10_ colony forming units per mL (log CFU/mL). Three replicates of each experiment were carried out.

#### 2.3.2. Physicochemical Analysis

Changes in levels of pH and temperature (ºC) in the process wash water during vegetable processing were determined. Product pH was also measured: 50 g of the vegetable sample were transferred to a stomacher bag containing 50 mL distilled water (1:1, w:v), homogenized by mixing for 100 s with a Bag-Mixer 400 mL stomacher, and the pH of the homogenate was measured at 23 °C using a pH meter with glass electrode (model GLP-21, Crison, Spain).

Free chlorine was measured using the N,N diethyl-p-phenylenediamine (DPD) colorimetric method (model HI95711, HANNA Instruments) with appropriate test kits. The chlorine dioxide concentration was measured by means of Dulcotest^®^ DT1B instrument (ProMinent, Spain) and peroxyacetic acid concentrations were measured using peroxyacetic acid strips (Quantofix, Macherey-Nagel, Germany).COD was determined by the standard photometric method using the Spectroquant PHARO 100 photometer (MERCK) and results were expressed as mg O_2_/L.

#### 2.3.3. Colour Analysis

A sphere spectrophotometer X-Rite (model SP64) was used for the instrumental color evaluation. Parameters L*, a* and b* will be determined according to the International Commission on Illumination (CIE, 1976), where: L* defines lightness (the maximum for L* is 100, which represents white and the minimum is 0, which represents black). A positive a* value indicates redness (−a* is greenness) and a positive b* values yellowness (−b* is blueness) on the hue-circle. a* and b* axes have no specific numerical limits. 

The instrument is calibrated to a standard white ceramic disk and a black trap before taking any measurements. Results are expressed as an average of several repetitions, related to D65 illuminant (standard daylight) with a standard observation angle of 10 degrees.

#### 2.3.4. Data Analysis

For each group of experiments, three replicate experiments were conducted. Microbial counts were transformed to logarithms before computing means and standard deviations; population densities were reported as log CFU/g for lettuce and carrot samples and as CFU/mL for process water samples. The General Linear Models (GLM) procedure of the Statistical Package for the Social Sciences (SPSS statistics 19) was applied. Significant differences between treatments with respect to bacterial reduction were analyzed by Tukey test at a level of significance of *p* ≤ 0.05.

## 3. Results and Discussion

### 3.1. Effect of Combined Treatments (Physical and Chemical Methods) on *E. coli* O157:H7Artificially Inoculated on Fresh-Cut Lettuce and Carrots

Once artificially inoculated, the initial concentrations of *E. coli* O157:H7 on fresh-cut lettuce and shredded carrots were 7.06 ± 0.21 and 6.45 ± 0.04 log CFU/g, respectively.

**Figure 1:**
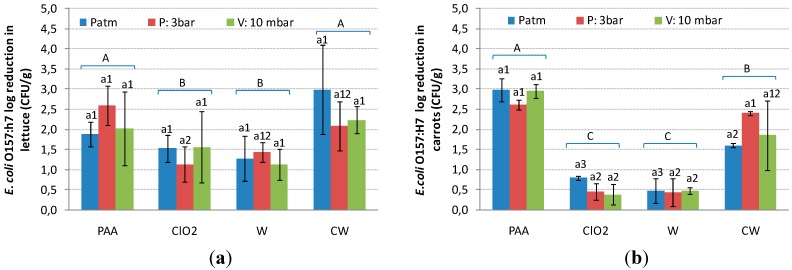
(**a**) *E. coli* reduction (log CFU/g before treatment - log CFU/g after treatment) in fresh-cut lettuce and (**b**) *E. coli* reduction in shredded carrots; after physical treatment (Patm: atmospheric pressure; P: positive pressure; V: negative pressure) in combination with PAA: peroxyacetic acid, ClO_2_: chlorine dioxide, W: tap water and CW: chlorinated water for 2 min.W and CW were used as controls. Bars indicate standard error of means (Within the same chemical agent, means sharing the same letter are not statistically different (*p* > 0.05); Within the same physical method, means sharing the same number are not statistically different (*p* > 0.05); Bars labeled with different capital letters indicate significant differences at *p* < 0.05.

The antimicrobial activity of different decontamination strategies on the population of *E. coli* on fresh cut vegetables (lettuce and carrot) is shown in Figure 1. 

In fresh-cut lettuce (Figure 1a), results suggested that the use of pressure conditions (in combination with sanitizers), did not significantly (*p* > 0.05) enhance the effectiveness of these decontamination technique compared with individual application of sanitizers at atmospheric conditions (Patm). Therefore, independently from the physical treatment performed, the average value of all the data for each sanitizing agent tested were calculated. Populations of *E. coli* were significantly decreased (*p* < 0.05) by 2.49 and 2.21 log CFU/g after washing with chlorine and peroxyacetic acid, whereas reductions of 1.41 and 1.28 log CFU/g were observed after washing with chlorine dioxide and tap water. These results are in accordance with most of the available literature regarding the use of sanitizers and leads to the conclusion that washing with water or with disinfectant solutions reduces the natural microbial populations on the surface of the produce by only 1 to 3 log units [16,17,18]. 

Washing inoculated shredded carrot (Figure 1b) under positive or negative pressures confirmed that washing in pressure conditions (P and V) had no significant (*p* > 0.05) effect on microbial reduction on the product surface. Regardless of the physical treatment, the average reduction attained by washing with tap water and chlorine dioxide was 0.46 and 0.54 log CFU/g, respectively, values significantly different (*p* < 0.05) from the reductions attained by PAA and chlorine. Peroxyacetic acid reduced *E. coli* O157:H7 populations by 2.84 log CFU/g, whereas the treatment with chlorine was able to reduce 1.95 log CFU/g.

The results obtained in this study indicate that peroxyacetic acid and chlorine have similar bactericidal power against *E. coli* O157:H7. Taking into account that the success of the combined strategy to completely remove the microorganisms present in fresh-cut vegetables was limited, the use of those disinfectants will be evaluated to maintain the microbial quality of wash water.

### 3.2. Effect of Combined Treatments (Physical and Chemical Methods) to Prevent *E. coli* O157:H7 Cross-Contamination on Process Wash Water

Washing contaminated fresh-cut vegetables in standardized washing water without sanitizers (W) resulted in a high contamination level in the washing water with *E. coli* from the washed product, as expected (Table 1). These results note that the use of tap water, without any sanitizing agent, is not enough to maintain water quality. Our results agree with other studies showing that the risk of cross-contamination is not removed by using large quantities of water [19,20]. Therefore, the use of effective disinfection sanitizers is strongly recommended to inactivate pathogens in the water used in the fresh-cut processing.

The use of PAA and CW showed the same efficacy in inactivating *E. coli* O157:H7 (below the limit of detection at all experimental conditions and in all types of vegetables) present in organically loaded processing wash water. This is in accordance with some authors [20,21] who found similar effectiveness of peroxyacetic acid and chlorine against *E. coli*. In the case of chlorine dioxide (ClO_2_) the dose used was not so effective in reducing the population of *E. coli* (1–2 log units were observed in the washing water after treatment with this chemical agent).

**Table 1:** *E. coli* O157:H7 (log CFU/mL) in processing water after washing fresh-cut lettuce and shredded carrots. ND: not detected (below the limit of detection of 1.0 CFU/mL).

	Washing Water of LETTUCE Processing			Washing Water of CARROT Processing		
	Patm	P: 3 bar	V: 10 mbar	Patm	P: 3 bar	V: 10 mbar
**PAA**	N.D.	N.D.	N.D.	N.D.	N.D.	N.D.
**ClO_2_**	1.53 ± 1.1	1.19 ± 0.7	1.79 ± 0.1	2.19 ± 1.9	2.81 ± 0.1	2.10 ± 0.6
**W**	5.34 ± 0.7	5.17 ± 0.2	5.54 ± 0.2	4.98 ± 0.6	4.88 ± 0.1	4.92 ± 0.1
**CW**	N.D.	N.D.	N.D.	N.D.	N.D.	N.D.

### 3.3. Effect of Combined Treatments (Physical and Chemical Methods) on Product Appearance 

**Figure 2:**
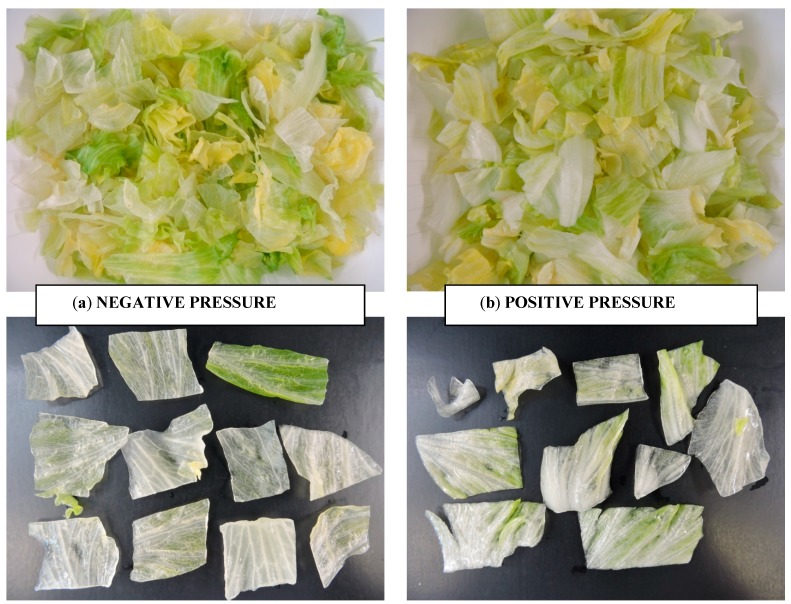
(**a**) Appearance of fresh-cut lettuce after combined processing (V + PAA) and detail of the lettuce tissue structure (**b**) Appearance of fresh-cut lettuce after combined processing (P + CW) and detail of the lettuce tissue structure.

Lettuce is a delicate product and, therefore, its textural quality can be affected by the application of positive/negative pressures. The response of fresh-cut lettuce to the vacuum/positive pressure processing, independent of the chemical sanitizer used, can be observed in Table 2 and Figure 2.

There were significant differences in L*, a*, b*, chroma and hue angle (*p* < 0.05) between fresh-cut lettuce before and after the treatment, with the exception of coordinate a* in the whiter leaves analyzed. Leaves before treatment showed higher L*, which is in accordance with the observation that lettuce samples before treatment are a lighter colour than samples after treatment. The decrease in b* parameter after the treatment means a less yellow-greenish colour than before the performance of decontamination procedure. This is partially due to the translucent appearance of lettuce, which reveals the black background used during the measurement.

**Table 2:** International Commission on Illumination (CIE) (L*a*b*) colour space (CIELAB) of fresh-cut lettuce, evaluated in two different areas of lettuce leaves (white and green part) and influenced by the physical treatment. Within the same column and type of leaf (white or green leaves), the asterisk indicates significant differences in the colorimetric parameters (*p* < 0.05).

		L*	a*	b*	C*	*h°*
***White Leaves***	Before treat.	67.2 ± 18.3*	−1.5 ± 0.4	4.8 ± 1.7*	5.0 ± 1.8*	107.7 ± 28.9*
	After treat.	42.3 ± 2.5	−1.5 ± 0.3	2.1 ± 1.2	2.5 ± 1.1	128.0 ± 15.0
***Green Leaves***	Before treat.	60.1 ± 1.8*	−8.5 ± 0.6*	27.1 ± 1.9*	28.4 ± 2.0*	107.3 ± 0.7*
	After treat.	41.6 ± 3.3	−7.0 ± 0.6	20.1 ± 3.1	21.3 ± 3.1	109.3 ± 1.8

Figure 2 showed that the application of positive/negative pressure affects the quality of lettuce due to its porous structure (it has many intercellular spaces enabling a large volume of air to be present among them). The application of high vacuum levels increased the porosity of vegetable tissue as a result of a high expansion and release of the gas inside the pores of the vegetable. Moreover, high vacuum level led to the removal of native liquid from the tissue structure conferring a translucent appearance to lettuce. Application of positive pressure also damaged the leaf tissue of lettuce although to a lesser extent than vacuum treatment.

According to the texture changes in fresh-cut lettuce, the contact area between sanitizers and vegetable tissue was significantly increased by means of the physical process. Nevertheless, the combination of technologies has little influence on the removal of *E. coli* O157:H7 from product surface, as was posed by the initial hypothesis of the study.

**Figure 3:**
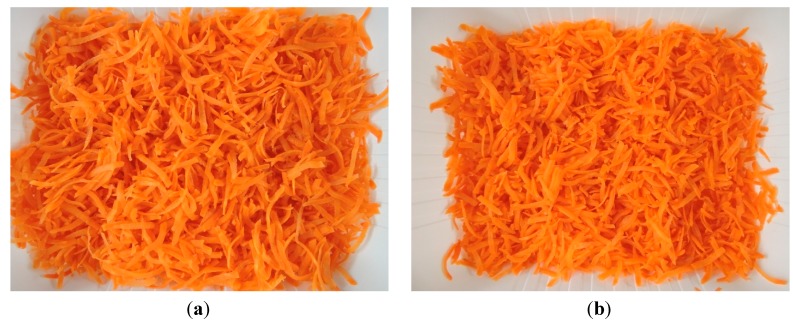
(**a**) Appearance of shredded carrot after combined processing (V + CW) (**b**) Appearance of shredded carrot after combined processing (P + PAA).

No changes in the appearance of shredded carrot were noticed with any of the treatments conducted. This is due to the densely packed cells of the tissue structure of carrots, which do not present intercellular spaces, showing a better performance in pressure conditions (Figure 3).

### 3.4. Effect of Combined Treatments (Physical and Chemical Methods) on Physicochemical Parameters 

The pH measurements for each treatment are given in Table 3. The effect of combined treatments on process water acidity depends on the type of sanitizer solution used. Whilst treatments with PAA significantly reduced (*p* < 0.05) the pH of process water compared to that of processing washing water without disinfectant, no differences (*p* > 0.05) were observed with the use of chlorine dioxide.

In general terms, regardless of the physical method used, the pH reduction in fresh-cut products were significant (*p* < 0.05) in PAA treatment compared with that observed after washing vegetables with tap water. The use of ClO_2_ did not lead to significant changes (*p* > 0.05) in the pH value of fresh-cut lettuce and carrots.

Another important issue to take into account is the COD contribution of the sanitizers. In this study, it was observed that the organic load of the processing water increased when PAA was added, while the addition of ClO_2_ had no effect (Table 4). The use of chlorine, by itself, did not affect the COD; however, when adjusting pH with citric acid there was also an increase in organic matter values.

**Table 3:** pH of tested solutions in processing wash water and in product (mean of all experiments ± standard deviation).

		pH_Lettuce Production Process		pH_Carrot Production Process	
		Washing Water	Product	Washing Water	Product
**PAA**	Patm	6.72 ± 0.1	6.45 ± 0.2	6.68 ± 0.0	6.41 ± 0.0
	P: +3 bar	6.65 ± 0.1	6.37 ± 0.1	6.55 ± 0.1	6.49 ± 0.3
	V: 10 mbar	6.90 ± 0.1	6.24 ± 0.2	6.89 ± 0.1	6.52 ± 0.2
**ClO_2_**	Patm	7.81 ± 0.1	6.69 ± 0.0	7.70 ± 0.1	6.69 ± 0.2
	P: +3 bar	8.07 ± 0.2	6.71 ± 0.0	7.68 ± 0.2	6.99 ± 0.2
	V: 10 mbar	7.97 ± 0.1	6.59 ± 0.1	7.76 ± 0.0	6.82 ± 0.2

**Table 4:** Chemical oxygen demand (COD) (mg O_2_/L) of process wash water in tested solutions at atmospheric conditions (mean of all experiments ± standard deviation). Within the same column, different letters indicate significant differences between sanitizing agents.

Sanitizers	COD (mg O_2_/L) in Process Wash Water	
	Lettuce	Carrot
**PAA**	483 ± 23.6^a^	1150 ± 28.3^a^
**ClO_2_**	262 ± 4.2^b^	969 ± 72.8^a^
**W**	254 ± 18.1^b^	947 ± 4.95^a^
**CW**	446 ± 74.6^a^	1125 ± 120.0^a^

## 4. Conclusions

In summary, experiments with combined disinfection methods were performed to evaluate the role of pressure conditions (positive and negative pressures) in the antimicrobial effectiveness of the washing process of fresh-cut vegetables. A synergistic effect was expected in the performance of the washing step by disrupting the hydrophobic bonding on leaf surfaces, allowing a greater penetration of the sanitizing agent.

The results obtained in this study demonstrate that the use of physical and chemical processes of disinfection, in a combined way, resulted in similar results to those of washing at atmospheric pressure (standard conditions). Hence, this strategy does not seem to be a promising alternative for improving decontamination efficiency in the sanitation of fresh-cut vegetables, as expected in the theoretical approach posed. In addition, these decontamination techniques have shown an indirect detrimental effect on fresh-cut product quality by affecting plant tissue physiology and structure.

However, when focusing the disinfection process towards the washing water rather than towards the product, PAA showed (in a short contact time) to be effective in inactivating bacterial pathogens in suspension in the washing water. Hence, PAA at a concentration of 100 mg/L is a promising alternative to chlorine, as a decontamination technique for minimally processed vegetables. Besides, it has the advantage of not being dependent on the presence of organic load in the solution, thus preservingits efficacy.

Applying ClO_2_ at concentration of 2 mg/L was insufficient for maintaining the microbial wash water quality in the washing process for both fresh-cut lettuce and shredded carrots. Further research should focus on the optimization of the use of this sanitizer in the production process of fresh-cut vegetables.

## References

[1] Van Haute S., Uyttendaele M., Sampers I. (2013). Organic acid based sanitizers and free chlorine to improve the microbial quality and shelf-life of sugar snaps. Int. J. Food Microbiol..

[2] Millán-Sango D., McElhatton A., Valdramidis V.P. (2015). Determination of the efficacy of ultrasound in combination with essential oil of oregano for the decontamination of *Escherichia coli* on inoculated lettuce leaves. Food Res. Int..

[3] Park S., Szonyi B., Gautam R., Nightingale K., Anciso J., Ivanek R. (2012). Risk factors for microbial contamination in fruits and vegetables at the preharvest level: A systematic review. J. Food Prot..

[4] Ölmez H., Kretzschmar U. (2009). Potential alternative disinfection methods for organic fresh-cut industry for minimizing water consumption and environmental impact. Lwt-Food Sci. Technol..

[5] Sapers G.M. (2001). Efficacy of washing and sanitizing methods for disinfection of fresh fruit and vegetable products. Food Technol. Biotechnol..

[6] Ölmez H., Temur S.D. (2010). Effects of different sanitizing treatments on biofilms and attachment of *Escherichia coli* and *Listeria monocytogenes* on green leaf lettuce. Lwt-Food Sci. Technol..

[7] Alvarado-Casillas S., Ibarra-Sanchez S., Rodríguez-García O., Martínez-Gonzáles N., Castillo A. (2009). Comparison of rinsing and sanitizing procedures for reducing bacterial pathogens on fresh cantaloupes and bell peppers. J. Food Prot..

[8] Ruiz-Cruz S., Acedo-Félix E., Díaz-Cinco M., Islas-Osuna M.A., González-Aguilar G.A. (2007). Efficacy of sanitizers in reducing *Escherichia coli* O157:H7, *Salmonella* spp. and *Listeria monocytogenes* populations on fresh cut carrots. Food Control.

[9] Velazquez L.D., Barbini N.B., Escudero M., Estrada C., De Guzman A.M.S. (2009). Evaluation of chlorine, benzalkonium chloride and lactic acid as sanitizers for reducing *Escherichia coli* O157:H7 and *Yersenia enterocolitica* on fresh vegetables. Food Control.

[10] Artés F., Gómez P., Aguayo E., Escalona V., Artés-Hernández F. (2009). Review: Sustainable sanitation techniques for keeping quality and safety of fresh-cut plant commodities. Postharvest Biol. Technol..

[B11-ijerph-12-08678] 11.BWBR0005758 Official title: Decision of December 10, 1992, Commodities Act Decree on the preparation and handling of food. Article 5. Netherlands Regulation (In Dutch).

[12] Vandekinderen I., Devlieghere F., De Meulenaer B., Veramme K., Ragaert P., Van Camp J. (2008). Impact of decontamination agents and a packaging delay on the respiration rate of fresh-cut produce. Postharvest Biol. Technol..

[13] Van Haute S., López-Gálvez F., Gómez-López V.M., Eriksson M., Devlieghere F., Allende A., Sampers I. (2015). Methodology for modeling the disinfection efficiency of fresh-cut leafy vegetables wash water applied on peracetic acid combined with lactic acid. Int. J. Food Microbiol..

[14] Monarca S., Richardson S.D., Feretti D., Grottolo M., Thruston A.D., Zani C., Navazio G., Ragazzo P., Zerbini I., Alberti A. (2002). Mutagenicity and disinfection by-products in surface drinking water disinfected with peracetic acid. Environ. Toxicol. Chem..

[15] Gómez-López V.M., Devlieghere F., Ragaert P., Debevere J. (2007). Shelf-life extension of minimally processed carrots by gaseous chlorine dioxide. Int. J. Food Microbiol..

[16] Keskinenn L., Burke A., Annous B. (2009). Efficacy of chlorine, acidic electrolyzed water and aqueous chlorine diovide solutions to decontaminate *Escherichia coli* O157:H7 from lettuce leaves. Int. J. Food Microbiol..

[17] Ongeng D., Devlieghere F., Debevere J., Coosemans J., Ryckeboer J. (2006). The efficacy of electrolysed oxidizing water for inactivating spoilage microorganisms in process water and on minimally processed vegetables. Int. J. Food Microbiol..

[18] Allende A., Martínez B., Selma M.V., Gil M.I., Suárez J.E., Rodríguez A. (2007). Growth and bacteriocin production by lactic acid bacteria in vegetable broth and their effectiveness at reducing *Listeria monocytogenes in vitro* and in fresh-cut lettuce. Food Microbiol..

[19] Holvoet K., Jacxsens L., Sampers I., Uyttendaele M. (2012). Insight into the prevalence and distribution of microbial contamination to evaluate water management in the fresh produce processing industry. J. Food Prot..

[20] López-Galvez F., Allende A., Selma M., Gil M.I. (2009). Prevention of *Escherichia coli* cross-contamination by different commercial sanitizers during washing of fresh-cut lettuce. Int. J. Food Microbiol..

[21] Veschetti E., Cutilli D., Bonadonna L., Briancesco R., Martini C., Cecchini G., Anastasi P., Ottaviani M. (2003). Pilot-plant comparative study of peracetic acid and sodium hypochlorite wastewater disinfection. Water Res..

